# Impact of prophylactic protamine administration following atrial fibrillation ablation on vascular and pericardial complications

**DOI:** 10.1093/europace/euag006

**Published:** 2026-04-13

**Authors:** Tess Calcagno, V Karthik, Bryan Baranowski, Mandeep Bhargava, Thomas Callahan, Mina K Chung, Roy Chung, Koji Higuchi, Ayman Hussein, Mohamed Kanj, Arshneel Kochar, Robert Koeth, Justin Lee, David Martin, Pasquale Santangeli, Tyler Taigen, Niraj Varma, Arwa Younis, Oussama M Wazni, Jakub Sroubek

**Affiliations:** Department of Internal Medicine, Cleveland Clinic, Cleveland, OH, USA; Department of Internal Medicine, Cleveland Clinic, Cleveland, OH, USA; Cardiac Electrophysiology Section, Department of Cardiovascular Medicine, Cleveland Clinic, 9500 Euclid Avenue, Cleveland, OH 44195, USA; Cardiac Electrophysiology Section, Department of Cardiovascular Medicine, Cleveland Clinic, 9500 Euclid Avenue, Cleveland, OH 44195, USA; Cardiac Electrophysiology Section, Department of Cardiovascular Medicine, Cleveland Clinic, 9500 Euclid Avenue, Cleveland, OH 44195, USA; Cardiac Electrophysiology Section, Department of Cardiovascular Medicine, Cleveland Clinic, 9500 Euclid Avenue, Cleveland, OH 44195, USA; Cardiac Electrophysiology Section, Department of Cardiovascular Medicine, Cleveland Clinic, 9500 Euclid Avenue, Cleveland, OH 44195, USA; Cardiac Electrophysiology Section, Department of Cardiovascular Medicine, Cleveland Clinic, 9500 Euclid Avenue, Cleveland, OH 44195, USA; Cardiac Electrophysiology Section, Department of Cardiovascular Medicine, Cleveland Clinic, 9500 Euclid Avenue, Cleveland, OH 44195, USA; Cardiac Electrophysiology Section, Department of Cardiovascular Medicine, Cleveland Clinic, 9500 Euclid Avenue, Cleveland, OH 44195, USA; Cardiac Electrophysiology Section, Department of Cardiovascular Medicine, Cleveland Clinic, 9500 Euclid Avenue, Cleveland, OH 44195, USA; Cardiac Electrophysiology Section, Department of Cardiovascular Medicine, Cleveland Clinic, 9500 Euclid Avenue, Cleveland, OH 44195, USA; Cardiac Electrophysiology Section, Department of Cardiovascular Medicine, Cleveland Clinic, 9500 Euclid Avenue, Cleveland, OH 44195, USA; Cardiac Electrophysiology Section, Department of Cardiovascular Medicine, Cleveland Clinic, 9500 Euclid Avenue, Cleveland, OH 44195, USA; Cardiac Electrophysiology Section, Department of Cardiovascular Medicine, Cleveland Clinic, 9500 Euclid Avenue, Cleveland, OH 44195, USA; Cardiac Electrophysiology Section, Department of Cardiovascular Medicine, Cleveland Clinic, 9500 Euclid Avenue, Cleveland, OH 44195, USA; Cardiac Electrophysiology Section, Department of Cardiovascular Medicine, Cleveland Clinic, 9500 Euclid Avenue, Cleveland, OH 44195, USA; Cardiac Electrophysiology Section, Department of Cardiovascular Medicine, Cleveland Clinic, 9500 Euclid Avenue, Cleveland, OH 44195, USA; Cardiac Electrophysiology Section, Department of Cardiovascular Medicine, Cleveland Clinic, 9500 Euclid Avenue, Cleveland, OH 44195, USA; Cardiac Electrophysiology Section, Department of Cardiovascular Medicine, Cleveland Clinic, 9500 Euclid Avenue, Cleveland, OH 44195, USA

**Keywords:** Atrial fibrillation ablation, Protamine, Heparin reversal, Vascular complications, Pericardial effusion, Bleeding complications, Hypotension

## Introduction

Protamine is frequently administered at the conclusion of atrial fibrillation (AF) ablation to reverse heparin and facilitate haemostasis, although objective evidence to support this practice is relatively sparse. Randomized data from an era predating routine use of vascular closure devices show that protamine significantly shortens the time to vascular haemostasis and ambulation after AF ablation. However, protamine was not shown to reduce the rate of vascular access complications or thromboembolic events.^1^ Furthermore, a retrospective study incorporating modern suture-based closure techniques has similarly not demonstrated differences in vascular complications or hospital stay with or without protamine.^2^

The 2023 ACC/AHA/ACCP/HRS guidelines and a 2024 EHRA/HRS/APHRS/LAHRS expert consensus statement recognize vascular complications as the most frequent adverse event after AF ablation, but these documents do not provide a specific recommendation on routine protamine use.^3,4^ Despite this, ∼70% of electrophysiologists report routine protamine administration.^5^ Protamine is generally well tolerated, but hypotension and arrhythmias have been described, and its routine prophylactic use may not confer meaningful benefit.^6^

## Methods

We retrospectively analysed all pulmonary vein isolation (PVI) procedures from January 2019 to June 2025, including combined PVI + left atrial appendage occlusion cases. Data were extracted from an institutional registry with complications adjudicated from structured documentation and full-text review.

Minimal or no interruptions were made to pre-procedural anticoagulation. Ultrasound guidance for vascular access was used in all cases per institutional policy. All cases used a continuous heparin infusion titrated to a goal activated clotting time (ACT) > 310 s. A decision whether to electively administer protamine to partially reverse ACT at the conclusion of the case was based on individual operator’s preference. If given, protamine was first administered as a 5–10 mg test dose over 10 min, followed by the total dose as a single bolus; total protamine dosing reflected operator preference. Cases where adverse events (including new pericardial effusions) occurred prior to case conclusion (i.e. situations where protamine may have been given reactively rather than prophylactically; *n* = 21) were excluded. Vascular closure was achieved in most cases using figure-of-8 suture and/or vascular closure devices. Manual pressure alone was used in 119 patients and was included in the non-vascular closure device group for analysis. Adverse outcomes included non-pericardial bleeding (retroperitoneal bleed, access-site haematoma, and pseudoaneurysm), pericardial effusion detected after the procedure, and hypotension reported after protamine administration. Access-site haematoma was defined as any documented haematoma requiring an intervention (including additional compression, imaging-guided evaluation, or treatment) and/or prolonged hospitalization (>2 nights), including cases that presented back to the emergency department after discharge. We reviewed discharge data for the subset of patients with available information.

Logistic regression models were performed unadjusted and adjusted for age modelled by decade of life, BMI, antiplatelet therapy, sex, and closure type (suture vs. vascular closure device). The data underlying this article will be shared on reasonable request to the corresponding author.

## Results

**Figure 1:**
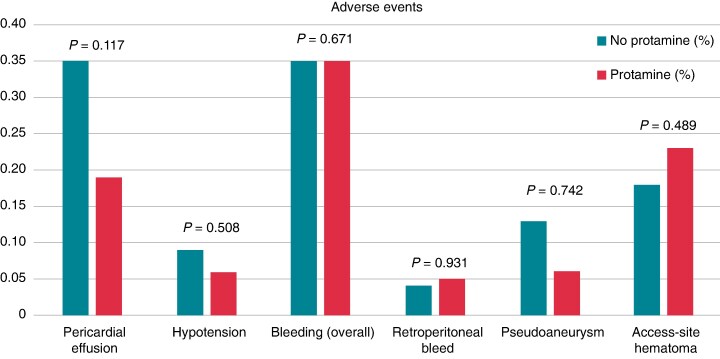
Adverse events by protamine use. Bar graph comparing the incidence of adverse events between patients who received protamine (2nd bar in each pair) and those who did not (1st bar in each pair). Events include pericardial effusion, post-operative hypotension, overall bleeding, retroperitoneal bleed, pseudoaneurysm, and access-site haematoma (without pseudoaneurysm). *P*-values are displayed above each comparison.

Among 11 668 ablation procedures, 9390 (80.3%) received protamine and 2278 (19.5%) did not. The median protamine dose was 28 mg (interquartile range 15–30 mg; point estimate based on a random subset of 100 patients). Baseline characteristics were similar in both groups (mean age 66.3 ± 9.9 vs. 67.2 ± 10.0 years; 31.7% vs. 32.2% female, *P* = 0.763). Closure with suture was more common in the protamine group (29.9% vs. 20.5%, *P* = 0.064). Event rates were low, and protamine use was not associated with worse outcomes. Pericardial effusion occurred in 0.19% with protamine vs. 0.35% without (adjusted OR 0.44, 95% CI 0.16–1.23, *P* = 0.177). Post-operative hypotension was infrequent (0.06% vs. 0.09%, adjusted OR 0.46, 95% CI 0.05–4.55, *P* = 0.508). Extramediastinal bleeding occurred in 0.35% of both groups (adjusted OR 0.67, 95% CI 0.25–1.84, *P* = 0.671); this included retroperitoneal bleeds (0.05% vs. 0.04%, *P* = 0.931), femoral pseudoaneurysms (0.06% vs. 0.13%, *P* = 0.742), and access-site haematomas (0.23% vs. 0.18%, *P* = 0.489) (*Figure 1*). No cases of protamine-related anaphylaxis were observed.

## Discussion

In this large, contemporary cohort, routine protamine administration after AF ablation did not reduce bleeding or pericardial complications. The low incidence of adverse events across both groups suggests that advances in mapping, ablation, and vascular closure techniques have substantially reduced bleeding risk, aligning with prior reports that found no outcome difference with protamine in the modern era.^2^

An earlier randomized study demonstrated that protamine could accelerate haemostasis and ambulation,^1^ but the clinical relevance of this finding may be limited following the advent of vascular closure techniques. Our findings are consistent with current guidelines, which do not endorse routine protamine reversal and highlight the absence of evidence linking it to reduced major complications.^3^

Although adverse haemodynamic events were not statistically different, rare but serious reactions, including hypotension and arrhythmia, have been reported and warrant caution.^7^ The high prevalence of prophylactic protamine use despite limited supportive evidence suggests that its use may be driven by institutional habit rather than necessity.

This analysis strengthens the argument that selective, rather than routine, protamine administration is appropriate in modern AF ablation practice. Future studies should define whether patient- or procedure-specific factors, such as sheath size or closure method, can guide targeted reversal strategies.

Our study has several limitations. Its retrospective, single-centre design may introduce unwanted confounders. Furthermore, our dataset did not include potentially important granular features, such as the end-procedural ACT values, the number and size of sheaths used in each case, or acute failure of vascular closure devices. Importantly, our study did not include any metrics reflecting post-procedural patient experience; it is possible that protamine use affected the duration of post-operative bedrest, rate of minor femoral bleeding, or other factors that may influence patient satisfaction. These limitations may affect the generalizability of our findings.

## Conclusions

In a registry of >11 000 AF ablations, routine protamine administration was not associated with reduced extramediastinal bleeding, pericardial effusion, or hypotension. Overall complication rates were low regardless of prophylactic reversal strategy, and no allergic reactions were observed. These findings suggest routine protamine reversal may not substantially impact periprocedural outcomes.

## Data Availability

The data that support the findings of this study are available upon reasonable request from the corresponding author.
